# Protocol for the adolescent hayfever trial: cluster randomised controlled trial of an educational intervention for healthcare professionals for the management of school-age children with hayfever

**DOI:** 10.1186/1745-6215-11-84

**Published:** 2010-08-05

**Authors:** Victoria S Hammersley, Samantha Walker, Rob Elton, Aziz Sheikh

**Affiliations:** 1 Allergy & Respiratory Research Group, Centre for Population Health Sciences, The University of Edinburgh, Medical School, Teviot Place, Edinburgh, EH8 9AG, UK; 2 Education for Health, The Athenaeum, 10 Church Street, Warwick, CV34 4AB, UK

## Abstract

**Background:**

Seasonal allergic rhinitis (hayfever) is common and can contribute to a considerable reduction in the quality of life of adolescents. This study aims to examine the effectiveness of standardised allergy training for healthcare professionals in improving disease-specific quality of life in adolescents with hayfever.

**Methods/Design:**

Adolescents with a history of hayfever registered in general practices in Scotland and England were invited to participate in a cluster randomised controlled trial. The unit of randomisation is general practices.

The educational intervention for healthcare professionals consists of a short standardised educational course, which focuses on the management of allergic rhinitis. Patients in the intervention arm of this cluster randomised controlled trial will have a clinic appointment with their healthcare professional who has attended the training course. Patients in the control arm will have a clinic appointment with their healthcare professional and will receive usual care.

The primary outcome measure is the change in the Rhinoconjunctivitis Quality of Life Questionnaire with Standardised Activities (RQLQ(S)) score between baseline and six weeks post-intervention in the patient intervention and control groups.

Secondary outcome measures relate to healthcare professionals' understanding and confidence in managing allergic rhinitis, changes in clinical practice, numbers of consultations for hayfever and adolescent exam performance.

A minimum of 11 practices in each arm of the trial (10 patients per cluster) will provide at least 80% power to demonstrate a minimal clinically important difference of 0.5 in RQLQ(S) score at a significance level of 5% based on an Intraclass Correlation Coefficient (ICC) of 0.02.

**Discussion:**

At the time of submission, 24 general practices have been recruited (12 in each arm of the trial) and the interventions have been delivered. Follow-up data collection is complete. 230 children consented to take part in the trial; however complete primary outcome data are only available for 160. Further recruitment of general practices and patients will therefore take place in the summer of 2010.

**Trial Registration:**

Current Controlled Trials ISRCTN95538067

## Background

In the UK, allergic diseases have an overall lifetime prevalence of about 30% in the general population, with a considerably higher prevalence in young people [1,2]. Intermittent allergic rhinitis (also known as seasonal allergic rhinitis or hayfever) affects up to 30% of adults and 40% of children at some time in their lives [2-5] Up to 80% of patients with asthma also have allergic rhinitis, and nearly 40% of those with allergic rhinitis have co-existing asthma [6,7]. Allergic rhinitis and its major co-morbidity asthma cause significant health burdens to the individual, and the impairment of quality of life experienced by patients with rhinitis is at least as severe as that of patients with asthma [8]. A recent editorial in *TheLancet *reported that the economic burden posed by allergic rhinitis has almost doubled since 2000 [9].

The Allergic Rhinitis in Asthma (ARIA) classification scheme was introduced in 2001 and reinforced in 2008; this subdivides allergic rhinitis into "intermittent" or "persistent" disease [8,10]. Previously, allergic rhinitis was subdivided based on time of exposure into "seasonal" (more commonly known as hayfever), "perennial" and "occupational" forms. Intermittent allergic rhinitis (IAR) is defined as symptoms being present for less than four days per week for less than four weeks and persistent allergic rhinitis refers to symptoms that are present for more than four days a week and for more than four weeks. This new ARIA classification has not yet been widely adopted in UK primary care, and patients with IAR are thus still given a Read code [11] for seasonal allergic rhinitis in their medical records and hence these terms and codes need to be searched when identifying local populations.

Common symptoms of allergic rhinitis include sneezing, itching, watery rhinorrhoea and nasal blockage, and these can have a considerable negative impact on children in terms of their physical, social and psychological well-being, and academic performance. Research has, for example, shown that children with allergic rhinitis indicated that they experienced particular problems with their schoolwork [12], and a recent case control study of teenage hayfever sufferers showed an association with an increased risk of unexpectedly dropping a grade in summer examinations (adjusted odds ratio 1.43; 95% CI 1.13-1.81) [13]. Children may also lose sleep, have reduced ability to concentrate, and have a risk of developing a major depressive disorder [14]. The achievement of optimal outcomes in children with allergic rhinitis depends on timely diagnosis, followed by implementation of measures to reduce allergen exposure, selection of safe and effective treatments and patient adherence to therapeutic regimens. This can be facilitated by appropriate training of healthcare professionals (HCPs) and patients in the optimum treatment choices, timing of medication commencement, appropriate techniques to ensure appropriate delivery of intranasal treatments and ensuring that compliance remains optimal.

Treatments for allergic rhinitis aim to minimise or eliminate symptoms, optimise quality of life and reduce the risk of developing co-morbidities. A report published in 2003 *Allergy - the unmet need*, commissioned by the Royal College of Physicians, clearly demonstrated current deficiencies in allergy services in the UK, and made a number of proposals to improve patient care, both in primary-and secondary-care. These findings have been confirmed in relation to allergic rhinitis in a UK survey of general practitioners whose practice had a self-declared interest in the management of allergic and respiratory disorders [15], which found considerable variation in their awareness and management of allergic rhinitis. This again suggests that an educational intervention covering all aspects of management of allergic rhinitis in primary care is both timely and important.

The House of Lords Science and Technology Committee report on Allergy (2006-7) recommended that the Department for Children, Families and Schools should review the clinical care that children receive at school, and should reassess the way they are supported through the examination season [16]. However, the knowledge and training in allergic diseases of teachers and support staff may not be sufficient to support children though this period. Enhancing the role of primary care professionals in ensuring control of allergy symptoms by improving training and engaging the patient in self-care may be a more beneficial and safer approach.

A multi-centre community based randomised controlled trial showed that standardised allergy education given to HCPs improved disease-specific quality of life in patients with perennial rhinitis [17]. This study found that a structured educational intervention was feasible to deliver in primary care, was well received by GPs and nurses, and improved outcomes in adults with perennial rhinitis. The present trial is building on this by testing the effectiveness of a modified version of this educational intervention in adolescents with hayfever.

### Aims of the study

The primary aim of this study is to examine the effectiveness of standardised allergy training in increasing disease-specific quality of life of adolescents with hayfever. A customised one-day short course, which focuses on allergic rhinitis and its main co-morbidity asthma, will be delivered to health care professionals.

The secondary aims are to examine whether attending an allergic rhinitis and asthma short course can enhance knowledge and skills of practitioners who consult with hayfever sufferers, changes in clinical practice, numbers of consultation for hayfever and adolescent exam performance.

### Specific objectives

1. To evaluate the effectiveness of standardised allergy training for HCPs on adolescent (12-18 years) rhinitis-specific quality of life.

2. To examine the impact of improving symptoms of hayfever on examination performance of adolescents.

3. To assess the change in allergy practice, improvement in confidence and understanding, and management of allergy symptoms of trained healthcare professionals.

## Methods/Design

### Trial design

We are conducting a pragmatic cluster randomised trial. Trial practices will receive either i) allergic rhinitis and asthma management training with support materials (rhinitis management algorithm and leaflet) or ii) support materials alone.

### Eligibility of general practices for entering the trial

#### Inclusion criteria

• General practices within the recruitment areas of the Scottish Primary Care Research Network (SPCRN) and the Northern and Yorkshire Research Network (NYREN).

• Practices that agreed to participate in the study and were willing to allow healthcare professionals to attend a one-day short course on allergic rhinitis and asthma.

#### Exclusion criteria

• Practices not interested in participating and/or unable to release practice staff to attend the training.

### Eligibility of patients for entering the trial

All young people aged 12-18 years with hayfever were eligible to participate. Hayfever was defined by the presence of a documented clinician diagnosis in the patient's health record and any evidence of treatment used for allergic rhinitis. Patients were excluded if they were unable to give consent or were taking part in any other clinical trials involving treatments for allergic rhinitis.

### Recruitment

#### General practices and health care professionals

We applied to SPCRN and NYREN for their assistance with practice recruitment. They wrote to general practices informing them of the study with an information flyer. Additional contact was made with general practices in Scotland via NHS Education for Scotland (NES) and local informal contacts. Where practices expressed an interest in participating, an information sheet was sent to each practice with the offer of a face-to-face or telephone discussion at which the study was explained in more details. A member of the practice team (Lead GP or Practice Manager) then signed a consent form if the practice decided to participate. Practices were asked to nominate a member of their team who regularly sees patients with hayfever, but who has not previously received postgraduate allergy training. Twenty four general practices were recruited.

#### Patient recruitment

In order to avoid the risk of allocation bias, practices were asked to identify all eligible patients aged 12-18 years, through searches of the practice electronic medical record, prior to randomisation. Patients with a recorded diagnosis of hayfever (Read code clinical terms v2: H17), and/or evidence of use of hayfever medication (oral antihistamines and topical steroids, drugs used in nasal allergy and topical nasal decongestants; Read code clinical terms v2: c8, c6, 18 and 19) were eligible to participate. The practices were asked to write to eligible participants sending an invitation letter with a participant information sheet, consent form and patient data collection form for return directly to the study team. Two versions of the consent form were used, one for 12-15 year old participants, which included a space for parental/guardian consent, and one for 16-18 year old participants. All recruitment materials were approved by Lothian 2 Research Ethics Committee. Reminder invitations were sent to non-responders by the practice nurse two weeks after the initial mailing. Consenting patients were asked to express their preferred method of communication with the research team: email, text messages or post.

### Intervention

The intervention was in two phases: the first phase was at the level of the practice/HCP and the second at the level of the patient.

#### HCP training

Those practices randomly allocated to the intervention arm were invited to nominate a HCP to attend an allergic rhinitis and asthma short course run by Education for Health. The short course was delivered by Education for Health trainers; the programme for this course is available in Additional file 1. Those practices randomised to the control arm received written information.

#### Appointment with HCP (patients in both groups)

Once a patient had consented to take part in the trial by returning the signed consent and data collection forms, they were invited by email, text message or phone to make an appointment with the nominated HCP by the research team. The research team liaised with the general practice to ensure all consenting patients had made an appointment during May-June 2009/10. Patients were seen by the HCP in their usual clinic setting. No guidance was given to either group about the format of the consultation.

### Allocation of trial interventions

The general practice was the unit of allocation. Randomisation to intervention or control was carried out separately within each of the five participating regions: Lothian; Borders; Durham and Tees Valley; Northumberland Tyne and Wear, York. For the four regions with more than two practices, this was done using minimisation based on achieving optimum balance for practice list size (three strata <5000, >5001, but <10000, >10000 patients currently registered) and deprivation score (Index of Multiple Deprivation), according to the methods described by Carter and Hood [18]

The reason for randomisation by centre was to help ensure an even distribution of intervention and control practices as there is likely to be a geographical variation in pollen counts between centres.

### Outcome measures

#### Primary outcome

• The change in the RQLQ(S) score between baseline and 6 weeks post-intervention in the intervention and control groups.

The RQLQ(S) measures the problems experienced by young people with hayfever. This is a validated and widely used tool in clinical trials [12,17,19,20]. It has been designed to ask patients about seven domains: activity; sleep; non-nose/eye symptoms; practical problems; nasal symptoms; eye symptoms; and emotional function.

Quality of life using the validated RQLQ(S) was measured at the beginning of the hayfever season (March 2009) prior to the HCP clinic appointment, and repeated at six weeks post-intervention.

### Secondary outcomes

• Weekly pollen count data collected over the duration of trial.

#### Clinical outcomes

• Patient reported symptom scores using a visual analogue scale.

• Overall assessment of hayfever symptoms compared with the previous season.

• Number of general practitioner and practice nurse consultations for hayfever, prescribed (from clinical records) and over-the-counter (from patients) medication data were collected.

#### Educational outcomes

Educational data will be collected via the Local Education Authority. This will be based on final grades for the age-specific assessment adjusted for pre-trial grades, where possible.

#### Process outcomes: assessment of change in clinical practice

These were measured using a questionnaire assessing change in allergy practice and improvement in confidence, understanding and management of allergic symptoms. All HCPs in the intervention arm were asked to complete this questionnaire immediately before and after the training, and after seeing their last patient in the trial.

### Sample size

There is little published literature about the likely design effect size in healthcare interventions and hayfever quality of life, however using data from a previous parallel group study using the RQLQ in adults with perennial rhinitis [17], a mixed-model analysis of variance gave an F-ratio of less than unity, indicating that there was less variation between practices than would be expected by chance (data available on request). This means that the inter-practice variance and hence the ICC is estimated as zero, and there would thus be no anticipated design effect for the proposed cluster randomised trial. There are however obvious differences between the study in adults and this cluster trial in adolescents, including the trial design; estimates of the between-practice variation in the proposed study and its effect on sample size are shown in Table 1.

**Table 1:** Number of patients needed with varying ICCs

ICC	Cluster size	No. of clusters	No. of patients
0.01	10	20	200

0.02	10	22	220

0.05	10	24	240

Sampsize [21] was used for sample size calculations. A cluster size of 10 was chosen as pilot work suggested that it should be possible to recruit at least this number of adolescents with diagnosed current hayfever from most general practices (data available on request).

Taking account of the cluster design, using a standard deviation of 1.2 [20] with a power of 80% to detect a minimal clinically important difference of 0.5 in RQLQ(S) score at a significance level of 5%, an estimate of the cluster size of 10 and an ICC of 0.02 required a total of 22 clusters and an adjusted sample size of 220 patients (unadjusted 180).

Based on these figures, we aimed to recruit at least 22 practices, inflated by 20% to account for possible losses to follow-up, resulting in 22 clusters (practices) recruiting 12 patients per practice, giving a total of 264 patients in the study (i.e. 132 per arm).

With these numbers, the study is sufficiently powered for the primary outcome measure; it is however likely to be underpowered for the secondary educational outcomes based on examination data [13].

### Compliance

All practices were given clear written information on what the study involved and were visited by the researcher prior to agreeing to take part in the study. In order to maximise the opportunities for intervention HCPs to attend the training, the course was run twice on separate dates in two different venues; all intervention HCPs attended a training day. The researcher assisted the practice with the mailings and reminders to eligible patients, and a clear account of payment for all practice time was given to the practices at the beginning, which included administrative time and backfill for the HCPs attending the training. The control group practices were offered the same short course at the end of the study and this was attended by nine out of the 12 control practices.

### Withdrawal of patients from the study

There were three points at which consenting patients could withdraw from the study:

i) prior to completing baseline questionnaires

ii) prior to attending the practice nurse appointment

iii) prior to completing the six weeks post-intervention questionnaires.

### Statistical analysis

Data analysis, using the following analysis plan, will be undertaken blind to the allocation arm. The primary analysis will be a per protocol analysis on complete cases only. An intention-to-treat analysis using the last observation carried forward is not feasible since imputing of the baseline data (which were collected before the pollen season) for subjects whose final RQLQ(S) is missing would not be a conservative assumption. This is because the lack of change from a value measured before the hayfever season might be better than expected. (i.e. baseline values may be low on a scale of 0-6 where 0 is not troubled and 6 is extremely troubled, and imputing these data for an RQLQ measured at the peak of the season, which may have been high might result in over-estimation of the intervention effect if more cases had missing data in the intervention group.)

### Descriptive analyses

#### Describing baseline characteristics of patients and practices

a) For each treatment arm, we will describe:

i) Patient age (mean and SD) and sex (number and percentage)

ii) Practice list size (median and IQR, or mean and SD if normally distributed)

iii) Practice population by age group (number and percentage)

iv) Practice deprivation (IMD or proxy measure)

v) Whole time equivalent GPs and practice nurses per practice (median and IQR, or mean and SD if normally distributed).

#### Comparison between treatment arms

The difference in the validated RQLQ(S) score between the intervention and control groups at baseline and six weeks post-intervention will be compared.

#### Adjusting for

1. Practice level stratum (region, list size and IMD or proxy) and baseline RQLQ(S).

2. Practice level stratum (region, list size and IMD or proxy). This will potentially be a more powerful comparison in the event that substantial numbers of subjects provide follow-up data, but not baseline data.

Multi-level modelling using a random-effects model will be used to take account of between and within cluster variation, adjusting for strata and individual covariates for example pre-intervention level of RQLQ(S). Estimates and standards errors of the intervention effects will be reported and normal chi-square tests on the ratio of these estimates to their standard errors will be used. An estimate and confidence intervals for the ICC will be calculated, adjusting for baseline RQLQ(S). Analysis will be undertaken using MLwiN.

### Missing data

#### RQLQ(S)

The RQLQ(S) is divided into seven domains, with varying numbers of questions per domain, and the overall RQLQ(S) score is calculated from the mean of each domain. Where responses to a whole domain within the RQLQ(S) are missing, this patient will be excluded from the analysis.

## Reporting and dissemination

Reporting will adhere to revised CONSORT criteria for cluster trials [22,23].

### Trial Steering Committee

The Trial Steering Committee (TSC) will monitor and supervise the trial and comment on any proposed amendments to the protocol. The TSC is chaired by Professor Anthony Avery and Dr Glenis Scadding, Dr Sarah Rodgers and Dr Sarah Armstrong are the other external members of the committee. The TSC has agreed to operate within the framework suggested in the MRC Guidelines for good clinical practice in clinical trials [24].

### Ethical considerations

The clinical trial will be conducted according to the Helsinki Declaration [25], Good Clinical Practice Guidelines [24] and NHS research governance requirements. Patients who have agreed to allow the study team to access their clinical and educational records have provided written informed consent. All patients were made aware that that can withdraw from the research at any time. The study has been approved by Lothian 2 Research Ethics Committee (Reference 08/S1102/37). All appropriate NHS Research and Development approvals have been obtained.

#### Study timeline

Trial Start: 1 August 2008

Baseline data collection: March 2009 and 2010

Interventions in general practice: April 2009 and 2010 (training), May/June 2009 and 2010 (patient appointments with HCPs)

End of interventions in general practice: June 2009 and 2010

End of 6 week follow-up: August 2009 and 2010

Start of data analysis: September 2010

Planned study end date: December 2010

Duration: 29 months

### Current study status

At the time of submission, outcome data has only been obtained for 160 patients from 24 general practices, leading to lower power than was originally intended for the study, and further recruitment is therefore planned in the summer of 2010. It is planned to recruit a further 10 practices, which will achieve the original target of 220 patients if cluster sizes are similar to those for 2009. We calculate that this will give similar power to that originally planned, because the gain in power from having more clusters of smaller size will be counterbalanced by some loss of power from the fact that there was substantial variation in the sizes of the different clusters.

## Abbreviations

ARIA: Allergic Rhinitis in Asthma; GP: General practice; HCP: health care professional; IAR: Intermittent allergic rhinitis; ICC: Intraclass correlation coefficient; NYREN: Northern and Yorkshire Research Network; RQLQ(S): Rhinoconjunctivitis Quality of Life Questionnaire with Standardised Activities; SAR: Seasonal allergic rhinitis; SPCRN: Scottish Primary Care Research Network.

## Competing interests

AS is a research advisor to Education for Health, SW is Director of Education & Research at Education for Health. All other authors declare no competing interests.

## Authors' contributions

AS, VH and SW conceived the study and together led the bid to secure funding for this work and manage the project. RE provided statistical and methodological advice in designing the study and all authors contributed to its implementation. VH was the researcher employed on this project and led the drafting of this paper. VH and AS are guarantors. All authors commented on draft versions, and read and approved the final manuscript.

## Supplementary Material

Additional file 1 **Programme for the Essential Asthma and Allergic Rhinitis Short Course**. Click here for file
