# Supplementary material for: Protocol for the adolescent hayfever trial: cluster randomised controlled trial of an educational intervention for healthcare professionals for the management of school-age children with hayfever
Source: Trials. 2010 Aug 5;11:84. doi: 10.1186/1745-6215-11-84 (PMC2922085; doi:10.1186/1745-6215-11-84)
Supplement: Additional file 1 — Programme for the Essential Asthma and Allergic Rhinitis Short Course. [file 1745-6215-11-84-S1.DOC]

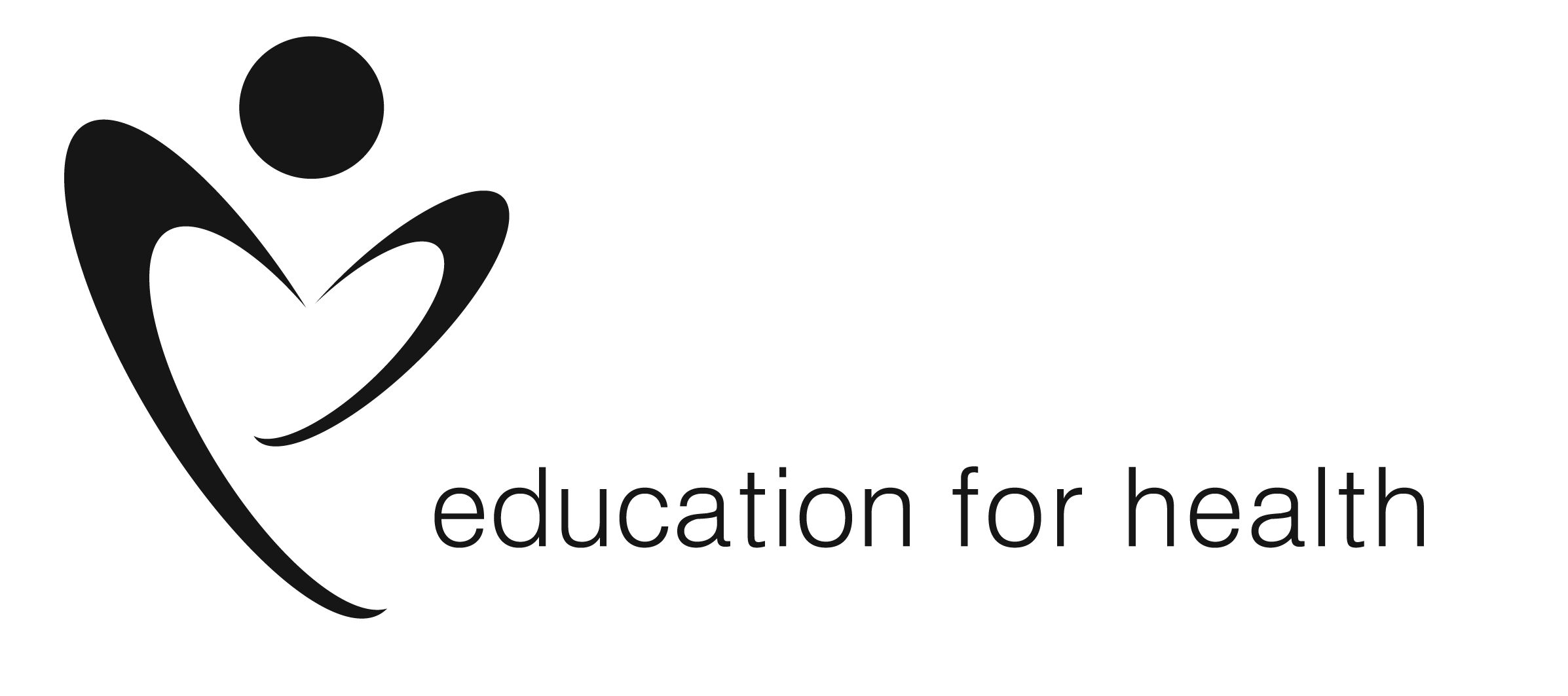
**Additional file 1**

Essential Asthma & Allergic Rhinitis Short Course

**PROGRAMME**

**9.00 am** REGISTRATION & COFFEE

**9.15 am Aims of the day and introductions**

**9.30 am An overview of asthma & rhinitis**

Impact on the individual and disease process

**10.30 am Diagnosis (1)**

History taking

Signs and symptoms, trigger factors

**11.00 am** COFFEE

**11.15 am Diagnosis (2)**

Objective diagnostic testing

Including reversibility, diurnal variation, skin prick tests and specific IgE.

**12.00pm Management of Asthma & Allergic Rhinitis**

Medications & Guidelines

To include new medications, sublingual immunotherapy and CFC free

**1.00 pm** LUNCH

**2.00 pm Management Continued**

Medications & Guidelines

**2.30 pm Device Workshop**

Practical demonstrations of inhalers and nasal sprays

Choosing the right device

**3.30 pm** TEA

**3.45 pm Putting it all together**

Case studies

**4.20 pm Summary and Evaluation**

**4.30 pm** CLOSE
